# LncRNA SAMD12-AS1 promotes cell proliferation and inhibits apoptosis by interacting with NPM1

**DOI:** 10.1038/s41598-019-48116-1

**Published:** 2019-08-12

**Authors:** Qi Liu, Ningning Liu, Qilin Shangguan, Fang Zhang, Wenjia Chai, Xiaomei Tong, Xin Zhao, Zhiwei Li, Dandan Qi, Xin Ye

**Affiliations:** 1 Key Laboratory of Pathogenic Microbiology and Immunology, Institute of Microbiology, Chinese Academy of Sciences (CAS), Beijing, 100101 China; 2 University of Chinese Academy of Sciences, Beijing, 100049 China; 3 302 Hospital of PLA, Beijing, 100039 China; 4 Savaid Medical School, University of Chinese Academy of Sciences, Beijing, 100049 China; 5 Institute for Ecological Research and Pollution Control of Plateau Lakes, School of Ecology and Environmental Science, Yunnan University, 650504 Kunming, China

**Keywords:** RNA, Long non-coding RNAs

## Abstract

Chronic hepatitis B virus infection is a major risk factor for hepatocellular carcinoma. HBV infection affects lncRNA expression in infected cells, but the detailed mechanism and biological significance are not yet clear. In this study, we focused on exploring the function of the HBV-upregulated lncRNA SAMD12-AS1 in cell proliferation. We found that there is a higher level of SAMD12-AS1 expression in tumors than in adjacent nontumorous liver tissues. We showed that ectopic expression of SAMD12-AS1 promotes cell growth and blocks apoptosis, while knockdown of SAMD12-AS1 inhibits cell proliferation and enhances etoposide-induced apoptosis. Using RNA immunoprecipitation and mass spectrometry, we determined that SAMD12-AS1 interacts with NPM1 and confirmed that SAMD12-AS1(1-350) is required for the interaction with NPM1. As it is known that NPM1 interacts with the E3 ligase HDM2 and reduces HDM2-mediated p53 degradation, we examined whether SAMD12-AS1 can affect p53 stability. Overexpression of SAMD12-AS1 caused a reduction in p53 protein levels by shortening its half-life. Conversely, knockdown of SAMD12-AS1 prolonged the half-life of p53. We further demonstrated that SAMD12-AS1 increased the interaction of HDM2 and p53 and enhanced p53 ubiquitination. Our findings reveal that HBV-upregulated SAMD12-AS1 regulates cell proliferation and apoptosis via the NPM1-HDM2-p53 axis.

## Introduction

Chronic hepatitis B (CHB) virus infection is the leading cause of hepatocellular carcinoma (HCC) and affects more than 250 million individuals^1,2^. Persistent HBV infection can cause cirrhosis and HCC, which has become a worldwide health problem^3^.

Previous studies indicate that HBV is involved in HCC development. HBV upregulates the transcription factors GATA2 and GATA3, which could suppress the expression of MICA/B to help hepatoma cells escape NK cell detection^4^. HBV inhibits TGF-β-induced apoptosis by upregulating Smad7 to downregulate TGF-β signaling and promote tumor development^5^. An HBV mRNA promotes tumor growth and cell invasion by sponging miR-122 to upregulate PBF (PTTG1 binding factor)^6^. Furthermore, HBV-encoded HBx promotes viral transcription by promoting the degradation of the Smc5/6 complex^7^.

Long noncoding RNAs (lncRNAs), which are defined as transcripts longer than 200 nucleotides without protein-coding function, have received increased attention as an increasing number of lncRNAs are identified^8–10^. It has been reported that lncRNAs play important roles in different processes. For instance, lncRNA NRON promotes HIV-1 latency by facilitating Tat ubiquitin-mediated degradation^11^. LncHIFCAR acts as a transcriptional coactivator for HIF-1α and promotes oral cancer development^12^. LncMyoD binds to IMP2 to inhibit IMP2-mediated translation of proliferation genes and regulate skeletal muscle differentiation^13^. LncRNAs play crucial roles in HCC development. LncRNA MVIH is associated with tumor growth and intrahepatic metastasis^14^. LncRNA HULC is overexpressed in HCC, which contributes to HCC progression by downregulating lipid metabolism through the RXRA signaling pathway^15^. LncRNA-hPVT1 stabilizes NOP2 to enhance cell proliferation as well as pluripotency of HCC cells^16^. LncRNA-DANCR was identified as a tumor-associated lncRNA by genome-wide analyses in two HCC cohorts, which greatly promoted stemness properties in HCC cells^17^. Recently, it has been reported that HBV-related lncRNAs contribute to HCC development. LncRNA-HEIH was identified as a highly expressed lncRNA in HBV-related HCC and acts as an oncogenic lncRNA that promotes tumor progression^18^. The HBV-encoded HBx suppresses the expression of lncRNA-Dreh, which functions as a tumor suppressor^8^. In addition, it has been found that HBx increases lncRNA UCA1 transcription to promote HCC progression^19^.

Previously, we analyzed differentially expressed lncRNAs between HBV-negative (HepG2) and HBV-positive (HepG2-4D14) cells and identified a series of HBV-associated lncRNAs, including lnc-HUR1, which promotes cell growth and tumorigenesis by inhibiting p53 transcriptional activity^20^. Here, we focused on lncRNA SAMD12-AS1. We show that HBV-encoded HBx enhances SAMD12-AS1 transcription. Functional analyses indicate that SAMD12-AS1 promotes cell proliferation and inhibits apoptosis. Further studies demonstrate that SAMD12-AS1 interacts with NPM1, which reduces its association with the E3 ligase HDM2 and consequently enhances the interaction of HDM2 and p53 to promote p53 degradation. Our study reveals that HBx-upregulated SAMD12-AS1 reduces p53 stability through the NPM1-HDM2-p53 axis, which in turn affects cell proliferation and apoptosis.

## Results

### HBV-encoded HBx enhances SAMD12-AS1 transcription

**Figure 1:**
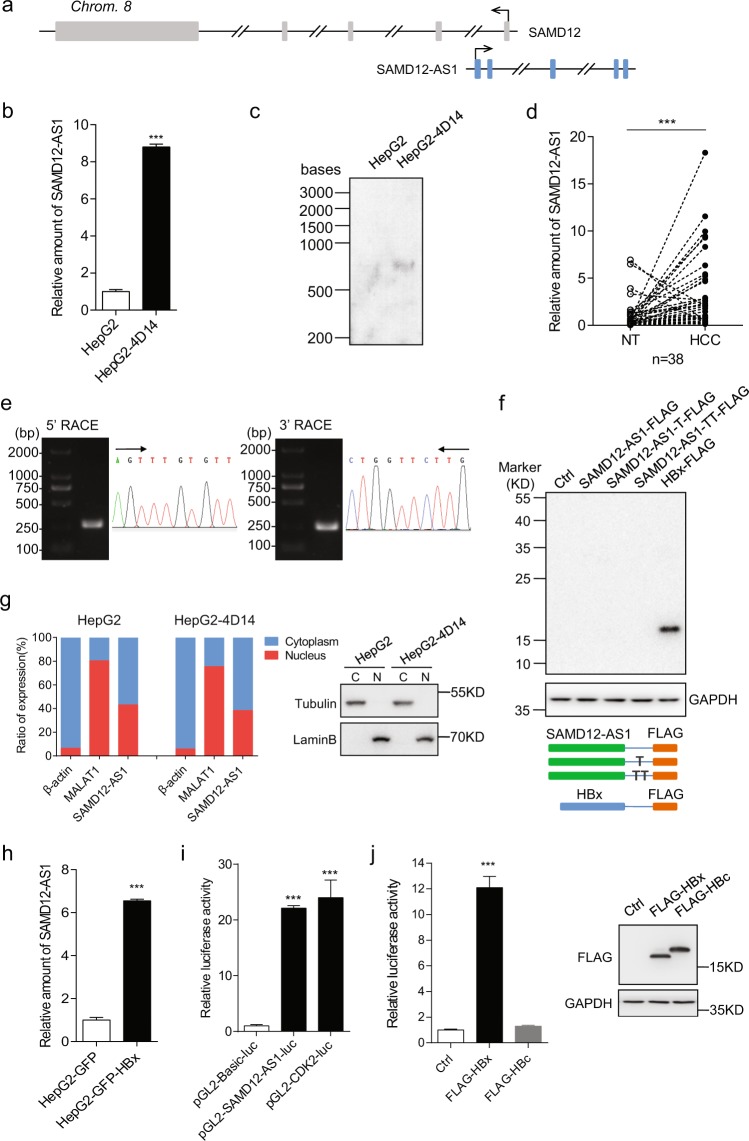
SAMD12-AS1 is upregulated by HBx. (**a**) Schematic map of the SAMD12 and SAMD12-AS1 genomic locus on chromosome 8. (**b**) Relative amounts of SAMD12-AS1 in HepG2 and HepG2-4D14 cells were examined by qRT-PCR. (**c**) Total RNA extracted from HepG2 and HepG2-4D14 cells was subjected to Northern blot with DIG-labeled DNA probe for SAMD12-AS1. (**d**) Total RNA from tumor tissues of HCC and corresponding adjacent normal tissues (NT) was extracted to measure SAMD12-AS1 levels using qRT-PCR. (**e**) RNA was extracted from HepG2 cells and subjected to 5′ and 3′ RACE assays for SAMD12-AS1. (**f**) Full-length SAMD12-AS1 was cloned into pcDNA3.1 in three open reading frames with a C-terminal FLAG tag. The plasmids were subsequently transfected into 293 T cells. After 48 h, the cell lysates were harvested and subjected to immunoblotting with anti-FLAG antibody. HBx-FLAG served as a positive control. (**g**) RNA from nuclear and cytoplasmic fractions of HepG2 and HepG2-4D14 cells was extracted and subjected to qRT-PCR to quantify SAMD12-AS1 normalized to β-actin mRNA and using MALAT1 as a control (left). The quality of cell fractionation was determined by immunoblotting with anti-Tubulin and anti-Lamin B antibodies. C, cytosolic fraction; N, nuclear fraction (right). (**h**) Total RNA was extracted from HepG2-GFP and HepG2-GFP-HBx stable cell lines, and the amount of SAMD12-AS1 was examined by qRT–PCR. (**i**) HepG2 cells were transfected with pGL2-Basic-luc, pGL-SAMD12-AS1-luc and pGL2-CDK2-luc as a positive control. The cell lysates were collected for a luciferase assay. The relative luciferase activities were calculated. (**j**) HepG2 cells were cotransfected with pGL-SAMD12-AS1-luc and pCMV FLAG-HBx, pCMV FLAG-HBc or empty vector as a control. The cell lysates were subjected to luciferase assay (left) and immunoblotting (right). ***P < 0.001. The data in b, h, i and j are representative of three independent experiments.

Previously, we identified 38 lncRNAs that were upregulated in HepG2-4D14 cells^20^. In this study, we focused on SAMD12-AS1, as it has been reported that SAMD12-AS1 is associated with neuroblastoma progression^21^. SAMD12-AS1 is located in the same locus as SAMD12 on chromosome 8 (Fig. 1a). qRT-PCR data showed that SAMD12-AS1 was upregulated in HepG2-4D14 cells compared with HepG2 cells, which is consistent with the RNA-Seq data^20^ (Fig. 1b). Northern blot data confirmed that SAMD12-AS1 is upregulated in HepG2-4D14 cells (Fig. 1c). We then examined SAMD12-AS1 expression in tumors and adjacent nontumorous tissues in 38 paired liver samples (Supplementary Table 1)and observed that the amount of SAMD12-AS1 in 32 tumor samples was higher than that in nontumorous tissues (Fig. 1d). We performed 5′ and 3′ RACE to determine the full sequence of SAMD12-AS1 (Fig. 1e). To confirm that SAMD12-AS1 is indeed a noncoding RNA, we generated C-terminally FLAG-tagged SAMD12-AS1 constructs in three reading frames and transfected the constructs into 293T cells. Immunoblotting indicated that SAMD12-AS1 cannot be translated (Fig. 1f). Analysis in ORF Finder (NCBI) and Coding Potential Assessment Tool (CPAT) also supported that SAMD12-AS1 is a noncoding RNA (Supplementary Fig. S1). Next, we analyzed the subcellular localization of SAMD12-AS1. As shown in Fig. 1g, SAMD12-AS1 localized in both the cytoplasm and the nucleus. Since HBx is a known transcriptional regulator, we compared the expression level of SAMD12-AS1 in HepG2-GFP and HepG2-GFP-HBx cells and found that the SAMD12-AS1 level in HepG2-GFP-HBx was significantly higher than that in HepG2-GFP (Fig. 1h). Using Promoter Scan and Neural Network Promoter Prediction, one region (−425 to 1) was predicted as the promoter of SAMD12-AS1. We generated a luciferase reporter for this region and performed a luciferase assay using the CDK2 promoter as a positive control. As shown in Fig. 1i, this region demonstrated high promoter activity. We then cotransfected HepG2 cells with the pGL2-SAMD12-AS1-luc reporter and pCMV FLAG-HBx or pCMV FLAG-HBc and observed that luciferase activity in HBx-expressing cells was over 10-fold higher than that in control cells (Fig. 1j). These results indicate that HBx can enhance the transcription of SAMD12-AS1.

### SAMD12-AS1 promotes cell proliferation and inhibits apoptosis

**Figure 2:**
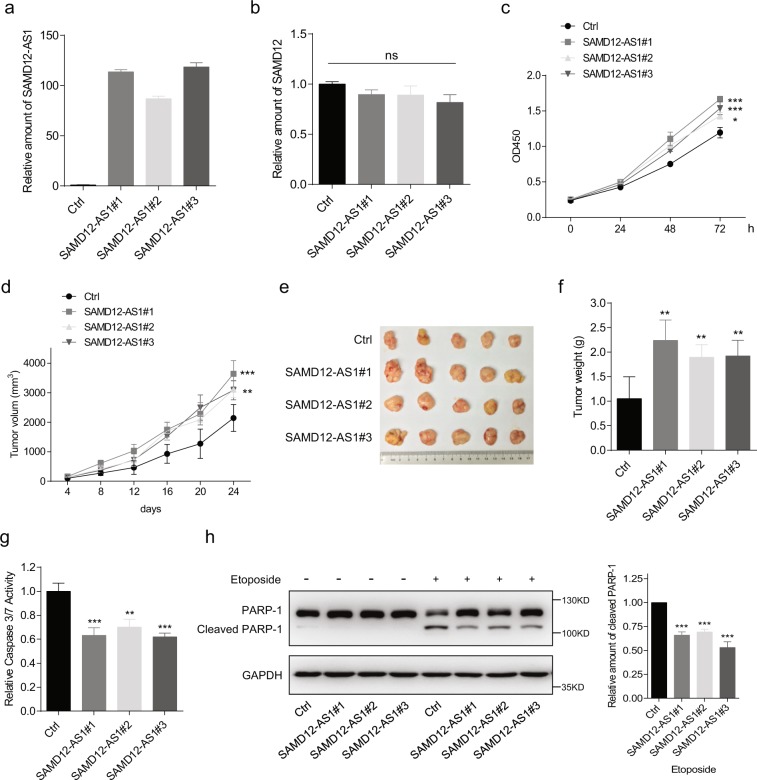
SAMD12-AS1 promotes cell growth and blocks apoptosis. (**a**) HepG2 cells were infected with lentivirus carrying SAMD12-AS1 or control virus. Three clones of HepG2 cells stably expressing SAMD12-AS1 (SAMD12-AS1#1, #2 and #3) were chosen, and the amount of SAMD12-AS1 was examined by qRT-PCR. (**b**) The relative amount of SAMD12 mRNA was examined by qRT-PCR in Ctrl and SAMD12-AS1 cell lines. (**c**) Growth curves of Ctrl and SAMD12-AS1 cells were determined using CCK-8 assays. (**d**–**f**) Ctrl and SAMD12-AS1 cells were injected into 6-week-old BALB/c nude mice (5 per group). Tumor sizes were measured at the indicated times (**d**). At day 24, the mice were sacrificed, and tumors were dissected out and photographed (**e**). Tumor weight was measured (**f**). (**g**,**h**) The above cells were treated with etoposide (2 μM) for 48 h. The cell lysates were subjected to caspase-3/7 activity assay (**g**) and immunoblotting with anti-PARP-1 antibody (h, left). The relative amount of cleaved PARP-1 was quantified using ImageJ (h, right). *P < 0.05; **P < 0.01; ***P < 0.001; ns, not significant. The data in (**a**–**c**,**g**,**h**) are representative of three independent experiments.

We generated HepG2 cell lines stably expressing SAMD12-AS1, (SAMD12-AS1#1, SAMD12-AS1#2, and SAMD12-AS1#3), and examined SAMD12 levels (Fig. 2a,b). No significant difference was observed in SAMD12 mRNA levels in HepG2 SAMD12-AS1 and control cells, suggesting that SAMD12-AS1 did not affect the transcription of SAMD12 mRNA. Next, we measured the proliferation of these cell lines and found that HepG2 SAMD12-AS1 cells grew faster than control cells (Fig. 2c). We also examined the growth of the above cells in nude mice. As shown in Fig. 2d–f, SAMD12-AS1 cells grew faster and developed larger tumors than control cells. We then treated cells with etoposide to induce apoptosis and analyzed caspase-3/7 activity. The data indicate that caspase-3/7 activity in SAMD12-AS1 cells was lower than that in control cells (Fig. 2g). Consistent with these findings, PARP-1 cleavage in HepG2 SAMD12-AS1 cells was significantly lower compared to that in control cells (Fig. 2h). These data indicate that SAMD12-AS1 promotes cell proliferation and inhibits apoptosis.

**Figure 3:**
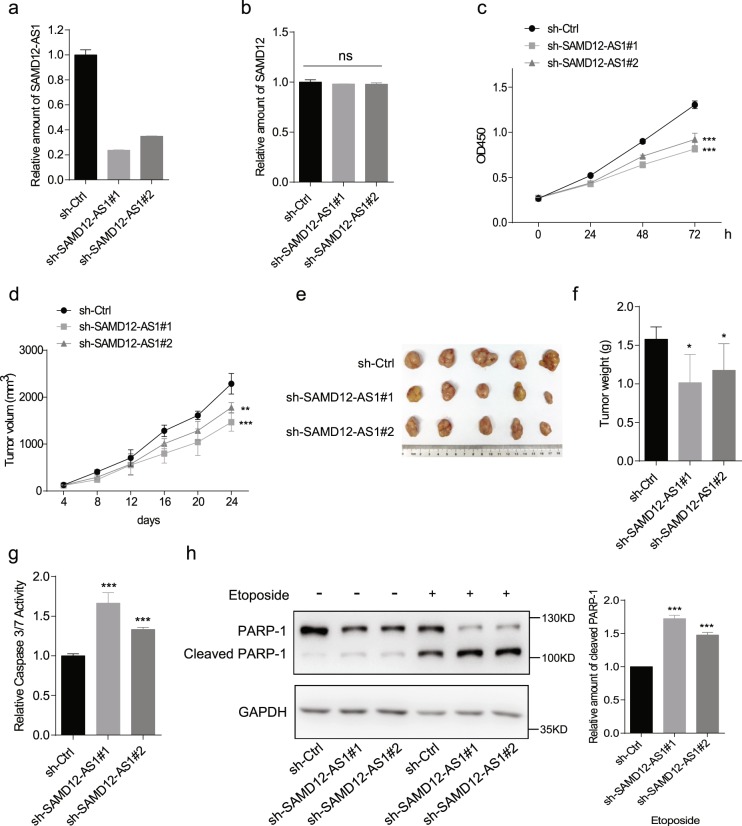
Knockdown of SAMD12-AS1 inhibits cell proliferation and promotes apoptosis. (**a**,**b**) Total RNA from HepG2 sh-Ctrl, sh-SAMD12-AS1#1 and sh-SAMD12-AS1 #2 cell lines was extracted and subjected to qRT-PCR to quantify the amount of SAMD12-AS1 (**a**) and SAMD12 mRNA (**b**). (**c**) Growth curves for the above cell lines were determined using CCK-8 assays. (**d**, **e**, **f**). HepG2 sh-Ctrl and sh-SAMD12-AS1 cell lines were injected into 6-week-old BALB/c nude mice (5 per group). Tumor sizes were measured at the indicated times (**d**). At day 24, the mice were sacrificed, and tumors were dissected out and photographed (**e**). Tumor weight was measured (**f**). (**g**,**h**) The indicated cells were treated with etoposide (2 μM) for 48 h. The cell lysates were subjected to caspase-3/7 activity assay (**g**) and immunoblotting with anti-PARP-1 antibody (h, left). The relative amount of cleaved PARP-1 was quantified using ImageJ (h, right). *P < 0.05; **P < 0.01; ***P < 0.001; ns, not significant. The data in a, b, c, g and h are representative of three independent experiments.

Next, we generated SAMD12-AS1 knockdown HepG2 cell lines (sh-SAMD12-AS1#1 and sh-SAMD12-AS1#2) using a lentiviral system (Fig. 3a) and examined SAMD12 mRNA levels. The results indicate that knockdown of SAMD12-AS1 did not affect transcription of SAMD12 mRNA (Fig. 3b). Growth curve data showed that cells with SAMD12-AS1 knockdown grew more slowly than control cells (Fig. 3c). Then, we examined whether knockdown of SAMD12-AS1 affects cell growth *in vivo*. The data showed that sh-SAMD12-AS1 cells grew more slowly and developed smaller tumors than control cells (Fig. 3d–f). Similar to the cells treated with etoposide, caspase-3/7 activity in SAMD12-AS1 knockdown cells was significantly higher than that in control cells (Fig. 3g). The relative amount of cleaved PARP-1 in SAMD12-AS1 knockdown cells was higher than that in control cells (Fig. 3h). These results indicate that SAMD12-AS1 knockdown inhibits cell proliferation and enhances etoposide-induced apoptosis.

### SAMD12-AS1 interacts with NPM1

**Figure 4:**
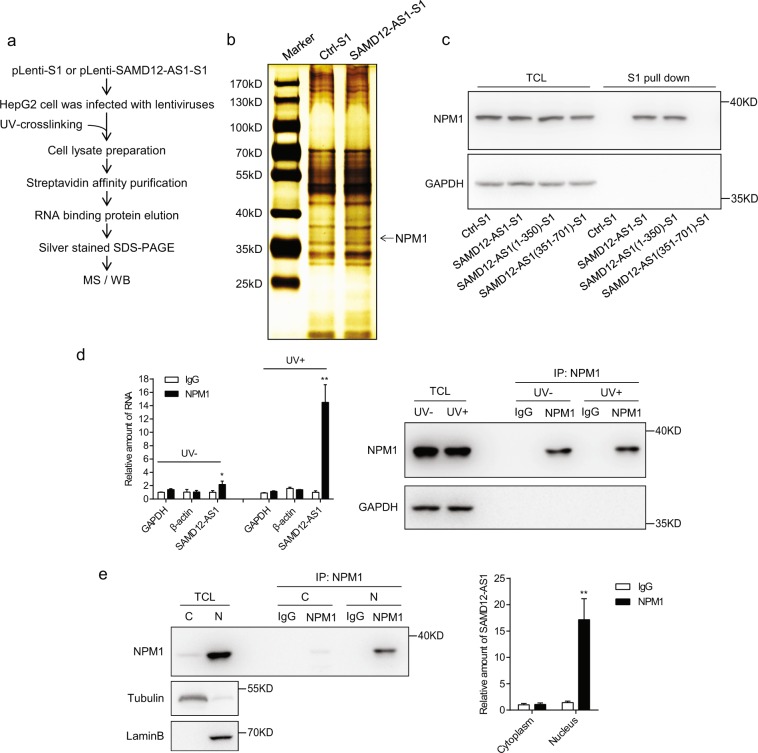
SAMD12-AS1 interacts with NPM1. (**a**) Schematic outline of the purification and identification of SAMD12-AS1-associated proteins. (**b**) The cell lysates from HepG2 Ctrl-S1 and SAMD12-AS1-S1 cells were subjected to pulldown with streptavidin beads. The eluted proteins were visualized with silver staining. (**c**) 293 T cells were transfected with full-length or truncated SAMD12-AS1 expression plasmids as indicated. The cell lysates were subjected to S1 pulldown followed by immunoblotting with anti-NPM1 and anti-GAPDH antibodies. (**d**) HepG2 cells with and without UV cross-linking were lysed and immunoprecipitated with anti-NPM1 antibody or rabbit IgG as a control, followed by qRT-PCR for SAMD12-AS1, with GAPDH and β-actin mRNA amplification as negative controls. (**e**) Cell lysates from nuclear and cytoplasmic fractions of HepG2 cells were immunoprecipitated with anti-NPM1 antibody or rabbit IgG as a control (left). The amount of SAMD12-AS1 bound to NPM1 was quantified by qRT-PCR (right). C, cytosolic fraction; N, nuclear fraction. *P < 0.05; **P < 0.01. The data in c, d and e are representative of three independent experiments.

To investigate the function of SAMD12-AS1, we used RNA immunoprecipitation and mass spectrometry to identify SAMD12-AS1-associated protein(s) (Fig. 4a). Briefly, we generated a HepG2 cell line in which SAMD12-AS1 with a 3′ S1 tag was stably expressed. The cell lysates were pulled down with streptavidin beads, and bound proteins were subjected to silver staining (Fig. 4b). Mass spectrometry indicated that NPM1 is a candidate interacting protein for SAMD12-AS1 (Supplementary Fig. S2). To confirm the interaction between SAMD12-AS1 and NPM1, we performed an S1 pulldown assay. As shown in Fig. 4c, SAMD12-AS1 and SAMD12-AS1(1-350) both interact with NPM1, while SAMD12-AS1(351-701) does not. Next, we immunoprecipitated NPM1 with and without UV cross-linking and detected NPM1-bound SAMD12-AS1. The data showed that SAMD12-AS1 was associated with NPM1 (Fig. 4d). Furthermore, to determine the subcellular localization of the SAMD12-AS1 and NPM1 complexes, we immunoprecipitated NPM1 from nuclear and cytoplasmic fractions (Fig. 4e, left), and quantified NPM1-bound SAMD12-AS1 (Fig. 4e, right). The data indicated that SAMD12-AS1 interacts with NPM1 mainly in the nucleus.

### SAMD12-AS1 reduces p53 protein by decreasing p53 stability

**Figure 5:**
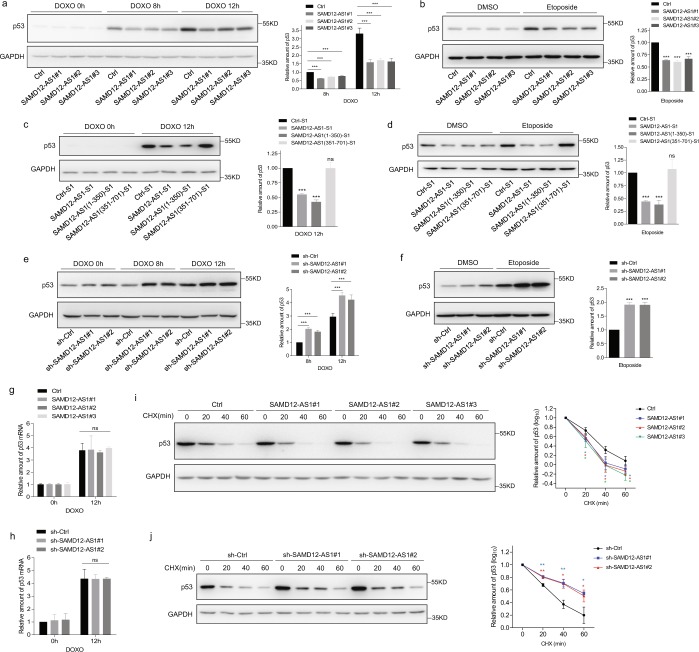
SAMD12-AS1 reduces p53 protein via decreasing p53 stability. (**a**,**b**) HepG2 Ctrl and SAMD12-AS1 cells were treated with doxorubicin (DOXO) (1 μg/ml) for the indicated times (0, 8 and 12 h) (**a**, left) and etoposide (4 μM) for 24 h (**b**, left). Cell lysates were harvested for immunoblotting with the indicated antibodies. The relative amount of p53 was quantified (**a**,**b**, right). (**c**,**d**) Cells were treated with DOXO (1 μg/ml) for 12 h (**c**, left) and etoposide (4 μM) for 24 h (**d**, left). Then, immunoblotting was performed with the indicated antibodies. The relative amount of p53 was quantified (**c**,**d**, right). (**e**,**f**) HepG2 sh-Ctrl and sh-SAMD12-AS1 cells were treated with doxorubicin (DOXO) (**e**, left) and etoposide (**f**, left). Cell lysates were harvested for immunoblotting with the indicated antibodies. The relative amount of p53 was quantified (**e**,**f**, right). (**g**,**h**) HepG2 Ctrl and SAMD12-AS1 cells (**g**) or HepG2 sh-Ctrl and sh-SAMD12-AS1 cells (**h**) were treated with DOXO (1 μg/ml) for 12 h. Total RNA was extracted and subjected to qRT-PCR to quantify p53 mRNA. (**i**,**j**) HepG2 Ctrl and SAMD12-AS1 cells (**i**, left) or HepG2 sh-Ctrl and sh-SAMD12-AS1 cells (**j**, left) were treated with CHX (25 μg/ml) for the indicated times (0, 20, 40 and 60 min). Cell lysates were harvested for immunoblotting with anti-p53 antibody. The relative amount of p53 was quantified on a log scale (**i**,**j**, right). The relative amount of p53 protein was quantified using ImageJ. The results are shown as the means ± S. D. from three independent biological replicates per group. *P < 0.05; **P < 0.01; ***P < 0.001; ns, not significant. The data are representative of three independent experiments.

**Figure 6:**
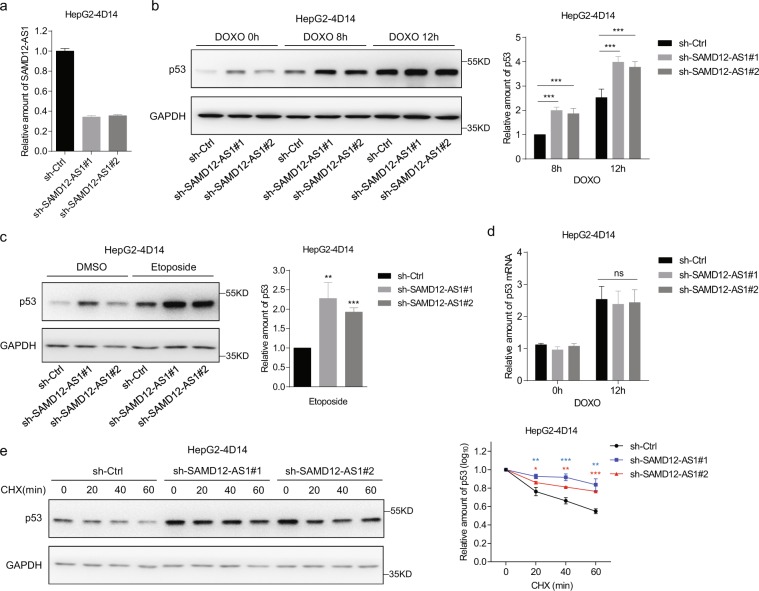
SAMD12-AS1 decreases p53 stability in HepG2-4D14 cells. (**a**) Total RNA from HepG2-4D14 sh-Ctrl and HepG2-4D14 sh-SAMD12-AS1 cell lines was extracted and subjected to qRT-PCR to quantify the amount of SAMD12-AS1. (**b**,**c**) Cells were treated with doxorubicin (DOXO) (1 μg/ml) (**b**, left) and etoposide (2 μM) (**c**, left). Cell lysates were harvested for immunoblotting with the indicated antibodies. The relative amount of p53 was quantified (**b**,**c**, right). d. HepG2-4D14 sh-Ctrl and HepG2-4D14 sh-SAMD12-AS1 cells were treated with DOXO for 12 h. Total RNA was extracted and subjected to qRT-PCR to quantify p53 mRNA. (**e**) Cells were treated with CHX (25 μg/ml) for the indicated times (0, 20, 40 and 60 min). Cell lysates were harvested for immunoblotting with anti-p53 antibody. The relative amount of p53 was quantified on a log scale (right). The relative amount of p53 protein was quantified using ImageJ. The results are shown as the means ± S. D. from three independent biological replicates per group. *P < 0.05; **P < 0.01; ***P < 0.001; ns, not significant. The data are representative of three independent experiments.

It has been reported that NPM1 interacts with the E3 ligase HDM2 and reduces HDM2-mediated p53 degradation. We wondered whether SAMD12-AS1 affects p53 stability through the NPM1-HDM2-p53 axis. First, we examined p53 protein levels in SAMD12-AS1 and control cells treated with DOXO (Doxorubicin) or etoposide. Immunoblotting showed that p53 protein levels in SAMD12-AS1 cells were significantly lower than those in control cells upon treatment with both DOXO and etoposide (Fig. 5a,b). We then performed a similar experiment with cells expressing truncated forms of SAMD12-AS1. The data indicated that p53 protein levels were reduced in HepG2 cells expressing full-length SAMD12-AS1 and SAMD12-AS1(1-350) but not SAMD12-AS1(351-701) (Fig. 5c,d), suggesting that NPM1-bound SAMD12-AS1 negatively regulates p53 levels. Conversely, the p53 protein level in SAMD12-AS1 knockdown cells was higher than that in control cells (Fig. 5e,f). We examined whether SAMD12-AS1 affects p53 mRNA levels by qRT-PCR. The data showed that overexpression of SAMD12-AS1 or knockdown of SAMD12-AS1 did not influence p53 mRNA levels (Fig. 5g,h). Next, we analyzed whether SAMD12-AS1 affects the half-life of p53 protein by treating cells with cycloheximide. The results showed that the degradation of p53 in SAMD12-AS1 cells was faster than that in control cells (Fig. 5i). Consistent with this result, the degradation of p53 in SAMD12-AS1 knockdown cells was slower than that in control cells (Fig. 5j). In addition, we performed similar experiments in HepG2-4D14 cells. The data showed that the p53 protein level in SAMD12-AS1-knockdown HepG2-4D14 cells was higher than that in control cells (Fig. 6a–c), while the p53 mRNA level was not changed (Fig. 6d). Furthermore, we found that p53 stability was decreased in SAMD12-AS1-knockdown HepG2-4D14 cells compared to control cells after CHX treatment (Fig. 6e). These results demonstrate that SAMD12-AS1 reduces p53 protein by affecting its stability.

### SAMD12-AS1 promotes HDM2-mediated ubiquitination of p53

**Figure 7:**
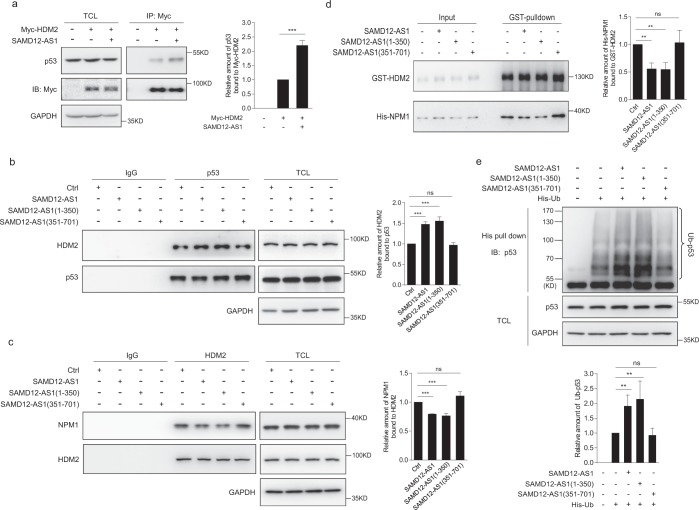
SAMD12-AS1 promotes HDM2-mediated ubiquitination of p53. (**a**) L02 cells were cotransfected with pCMV Myc-HDM2 and control plasmid or SAMD12-AS1-expressing plasmids. Cell lysates were subjected to immunoprecipitation followed by immunoblotting with the indicated antibodies (left). The amount of p53 bound to Myc-HDM2 was quantified (right). (**b**,**c**) L02 cells were transfected with control, SAMD12-AS1, SAMD12-AS1(1-350) or SAMD12-AS1(351-701) plasmids. Cell lysates were subjected to immunoprecipitation with anti-p53 antibody (**b**, left) or anti-HDM2 antibody (**c**, left), followed by immunoblotting with antibodies against HDM2, p53 or NPM1 as indicated. The relative amount of HDM2 bound to p53 and NPM1 bound to HDM2 were quantified (**b**,**c**, right). (**d**) GST-HDM2 and His-NPM1 were mixed with *in vitro* transcribed SAMD12-AS1, SAMD12-AS1(1-350) or SAMD12-AS1(351-701) and then subjected to pulldown with glutathione beads followed by immunoblotting with anti-GST and anti-His antibodies (left). The amount of His-NPM1 bound with GST-HDM2 was quantified (right). (**e**) L02 cells were cotransfected with pCMV His-Ub and control plasmid or SAMD12-AS1, SAMD12-AS1(1-350) or SAMD12-AS1(351-701)expression plasmids. Then, cells were treated with MG132 for 6 h, and cell lysates were subjected to His pulldown and immunoblotted with anti-p53 antibody (upper panel). The relative amount of ubiquitinated p53 (Ub-p53 in short) was quantified (lower panel). The relative amounts of p53, HDM2, NPM1, His-NPM1 and Ub-p53 were quantified using ImageJ. **P < 0.01; ***P < 0.001; ns, not significant. The data are representative of three independent experiments.

As we observed that SAMD12-AS1 promotes p53 degradation, we hypothesized that SAMD12-AS1 may bind NPM1 competitively and enhance the interaction of HDM2 and p53, which in turn would promote p53 ubiquitination. Therefore, we examined whether SAMD12-AS1 can enhance the interaction of HDM2 and p53. Since the transfection efficiency of HepG2 cells is too low, we performed the following experiments using the human hepatic cell line L02, which has a higher transfection efficiency than HepG2 cells. We transfected L02 cells with Myc-HDM2-expressing plasmid with or without pLenti-SAMD12-AS1. The cell lysates were immunoprecipitated with anti-Myc antibody and then immunoblotted with anti-p53 antibody. The data showed that the amount of HDM2-bound p53 was higher in SAMD12-AS1-expressing cells than in control cells (Fig. 7a). Then, we transfected the L02 cells with plasmids expressing SAMD12-AS1, SAMD12-AS1(1-350), and SAMD12-AS1(351-701). The cell lysates were collected for immunoprecipitation with anti-p53 antibody followed by immunoblotting with anti-HDM2 antibody. The data showed that the amount of p53-bound HDM2 in SAMD12-AS1- and SAMD12-AS1(1-350)-expressing cells was greater than that in control cells or SAMD12-AS1(351-701)-expressing cells (Fig. 7b). We also examined whether SAMD12-AS1 affects the interaction of NPM1 and HDM2. As shown in Fig. 7c, overexpression of SAMD12-AS1 and SAMD12-AS1(1-350) but not SAMD12-AS1(351-701) reduced the amount of NPM1 associated with HDM2. We then performed a GST pulldown assay using *E*.*coli*-expressed GST-HDM2 and His-NPM1 and SAMD12-AS1, SAMD12-AS1(1-350) or SAMD12-AS1(351-701) transcribed *in vitro*. The data indicated that SAMD12-AS1 and SAMD12-AS1(1-350) reduced the interaction of NPM1 and HDM2 (Fig. 7d). These data suggest that SAMD12-AS1 facilitates the interaction between HDM2 and p53 by blocking the association of NPM1 with HDM2. Next, we examined whether SAMD12-AS1 can enhance p53 ubiquitination. We transfected L02 cells with pCMV His-Ub and pLenti-SAMD12-AS1 or its truncated mutants and then treated the cells with MG132. The cell lysates were subjected to a His-pulldown assay. The data showed that the intensity of ubiquitinated p53 in SAMD12-AS1- and SAMD12-AS1(1-350)-expressing cells was higher than that in control cells and SAMD12-AS1(351-701)-expressing cells (Fig. 7e). These results demonstrate that SAMD12-AS1 enhances the interaction between HDM2 and p53 and promotes p53 ubiquitination and turnover.

**Figure 8:**
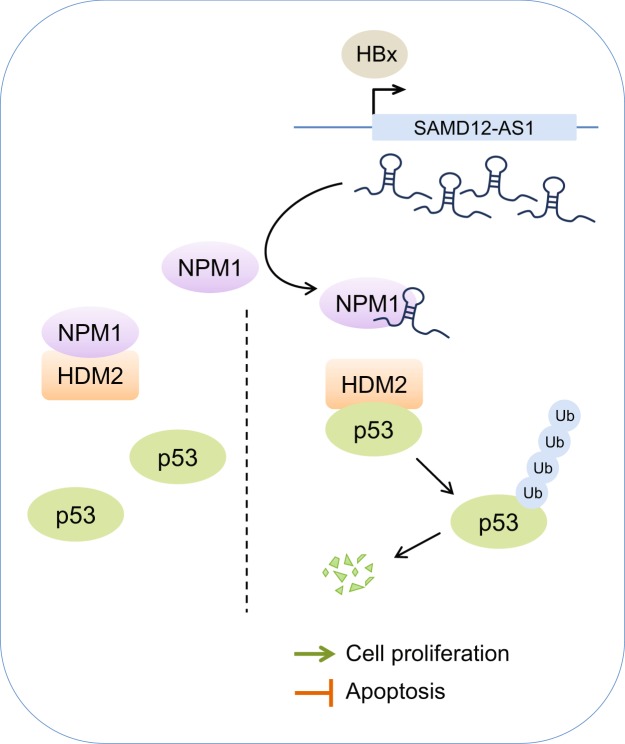
Schematic map of SAMD12-AS1 regulating cell proliferation and apoptosis. HBV-encoded HBx promotes the transcription of SAMD12-AS1. SAMD12-AS1 interacts with NPM1 to prevent its association with HDM2. Consequently, HDM2 binds to p53 and enhances p53 ubiquitination and degradation, which in turn promotes cell proliferation and inhibits apoptosis.

In summary, we identified that SAMD12-AS1 as a novel lncRNA upregulated by HBV HBx. We demonstrated that SAMD12-AS1 promotes cell growth and blocks apoptosis of hepatocytes. Furthermore, we found that SAMD12-AS1 interacts with nucleophosmin NPM1 and enhances HDM2-mediated p53 ubiquitination and degradation, consequently reducing p53 stability (Fig. 8). Our studies reveal the mechanism by which HBV regulates SAMD12-AS1 expression and a novel function of SAMD12-AS1 in cell proliferation and apoptosis.

## Discussion

The recent application of RNA-Seq to cancer transcriptomes has revealed an increasing number of lncRNAs associated with cancer development^22,23^. These lncRNAs have been found to participate in various aspects of cellular processes, such as cell growth, apoptosis, or genomic stability^24–27^. However, the detailed mechanisms by which lncRNAs regulate cell proliferation and tumorigenesis require further investigation.

Hepatitis B virus infection has been considered to be closely correlated with the development of hepatocellular carcinoma (HCC). Previous studies revealed that HBV HBx is a transcriptional regulator that regulates the expression of many genes. Recently, it has been reported that HBx also affects the transcription of lncRNAs^28^. For example, HBx downregulates lncRNA-Dreh, which promotes HCC development^8^. Furthermore, HBx could upregulate MALAT1, which promotes HCC development and metastasis by upregulating the expression of LTBP3^29^. Our current work revealed that HBx enhances lnc-HUR1 transcription, which interacts with p53 directly and interferes with p53 transcriptional activity^20^. In this study, we demonstrate that HBx-upregulated SAMD12-AS1 interacts with NPM1 and competes with the interaction of NPM1 with the E3 ligase HDM2, which causes a reduction in p53 stability and consequently promotes cell proliferation and tumor growth. These findings indicate that HBx promotes HCC development by influencing the protein expression and transcription of lncRNAs, thus providing the possibility of crosstalk between proteins and lncRNAs during HBV-associated HCC development.

It is known that NPM1 not only plays an important role in regulating rDNA transcription but also controls p53 stability by interacting with HDM2^30,31^. However, there is no report of an lncRNA regulating the NPM1-HDM2-p53 axis. Here, we provide evidence to show that SAMD12-AS1 interacts with NPM1 and enhances the interaction of HDM2 and p53, which in turn promotes the ubiquitin-mediated degradation of p53. Since p53 is identified as a tumor suppressor that is deregulated in various types of tumors, the negative correlation between SAMD12-AS1 and p53 stability implies that SAMD12-AS1 could be a prognostic marker for HCC and other types of tumors.

In addition to SAMD12-AS1, we identified a set of lncRNAs differentially expressed in HBV transgenic cells. Further studies investigating the functions of these lncRNAs will be important to aid our understanding of HBV-associated HCC development and may provide novel lncRNA targets to prevent and treat HCC.

## Methods

### Plasmids and reagents

Plasmids were constructed as follows: the SAMD12-AS1 and SAMD12-AS1-S1, SAMD12-AS1(1-350)-S1 and SAMD12-AS1(351-701)-S1 tagged genes were cloned into a lentiviral plasmid, pLentiLox3.7; the SAMD12-AS1, SAMD12-AS1-T, SAMD12-AS1-TT, and HBx genes were cloned into pcDNA3.1 with a C-terminal FLAG tag.; and pGL2-SAMD12-AS1-luc was constructed by cloning the predicted SAMD12-AS1 promoter (−425 to 1, genome location: nt118620575-nt118621000) into the pGL2-Basic vector.

Rabbit anti-NPM1 antibody was produced by immunizing rabbits with *E*. *coli*-expressed NPM1. Mouse anti-FLAG antibody (M2), rabbit anti-Myc antibody (9E10) and anti-Myc beads were purchased from Sigma Aldrich, USA. Mouse anti-GAPDH antibody, goat anti-human lamin B antibody, and anti-p53 antibody were purchased from Santa Cruz Biotechnology, USA. Rabbit anti-human tubulin antibody and rabbit anti-human poly(ADP-ribose) polymerase (PARP) antibody were purchased from Cell Signaling Technology, USA. Rabbit anti-HDM2 antibody was purchased from Bioworld, USA. HRP-conjugated secondary antibodies were purchased from Jackson Laboratory, USA. Anti-Mouse IgG (Heavy Chain)-HRP, anti-Mouse IgG (Light Chain)-HRP, anti-Rabbit IgG (Light Chain)-HRP, and anti-Rabbit IgG (Heavy Chain)-HRP were purchased from EASYBIO, China. The anti-HA-Tag beads were purchased from Abmart, and Ni-NTA beads were purchased from Qiagen, Germany.

### Cell culture and tumor samples

Human hepatoma cell line (HepG2), human embryonic kidney cell line (HEK293T) and human normal liver cell line (L02) were purchased from ATCC (Manassas, VA, USA). The HepG2-4D14 cell line, in which the HBV genome was stably integrated, was kindly provided by Dongping Xu (302 Hospital of PLA, China)^5^. All cell lines were cultured in DMEM supplemented with 10% fetal bovine serum at 37 °C and 5% CO_2_.

To generate SAMD12-AS1 overexpression cell lines, HepG2 cells were infected with lentivirus carrying SAMD12-AS1 or empty plasmid as a control. Then, GFP-positive cells were sorted, and cell clones stably expressing SAMD12-AS1 were chosen and named SAMD12-AS1#1, SAMD12-AS1#2 and SAMD12-AS1#3.

To generate S1-tagged SAMD12-AS1 and truncated SAMD12-AS1 overexpression cell lines, HepG2 cells were infected with lentivirus carrying SAMD12-AS1-S1, SAMD12-AS1(1-350)-S1, SAMD12-AS1(351-701)-S1 or empty plasmid as a control. Then, GFP-positive cells were sorted and named SAMD12-AS1-S1, SAMD12-AS1(1-350)-S1 and SAMD12-AS1(351-701)-S1, respectively.

To generate SAMD12-AS1 stably silenced HepG2 cells or HepG2–4D14 cells, HepG2 cells or HepG2-4D14 cells were infected with lentivirus carrying shRNAs (sh-SAMD12-AS1#1 and #2) targeting SAMD12-AS1. Then, GFP-positive cells were sorted. The shRNA-targeted regions are located at SAMD12-AS1 nt 451–469 (sh-SAMD12-AS1#1) and nt 641–659 (sh-SAMD12-AS1#2).

Liver tissues from 38 patients with HCC were collected from the 302 Hospital of PLA. The clinical characteristics of the patients are listed in Supplementary Table 1. Written informed consent was provided by all study participants. Patient samples were assigned arbitrary identification numbers based on the order of enrollment. The study protocol was approved by the ethics committee of the 302 Hospital of PLA^20^. All experiments were performed according to approved guidelines and regulations.

### Quantitative RT-PCR (qRT-PCR)

qRT-PCR was performed as previously published^6^. In brief, total RNA was extracted from cultured cells or tissues following standard TRIzol protocol (Invitrogen, USA). Then, reverse transcription was performed using cDNA Synthesis SuperMix (TransGen Biotech, China), and real-time PCR was performed with a SYBR green real-time PCR kit (Toyobo, Japan) according to the manufacturer’s protocol. Relative expression was quantified using the comparative threshold cycle (CT) method. Primers are listed below: SAMD12-AS1-F, 5′-CGTCTCTCCAAAGCAACTGAA-3′, SAMD12-AS1-R, 5′-CTTGAACTCCAGCAACTCTAGTC-3′; SAMD12-F, 5′-GTGCCTGACCAGAAA GGAACTC-3′, SAMD12-R, 5′-TTCTTCAACCACTTGCAGACATC-3′; p53-F, 5′-ACTGCCTTCCGGGTCACTGC-3′, p53-R, 5′-GTCAGTGGGGAACAAGAAGTGG AG-3′. All data were normalized to β-actin.

### Caspase activity assay

Cells were plated in 96-well plates and treated with etoposide (2 μM) for 48 h. The cell lysates were harvested to detect caspase activity using Caspase-Glo3/7 reagents (Promega, USA). Then, the luminescence of all samples was measured with a luminometer.

### Immunoblotting

Immunoblotting was performed as previously described^32^. Briefly, cell lysates were subjected to SDS-PAGE and blotted with the indicated antibodies. Then, the membranes were incubated with horseradish peroxidase-conjugated secondary antibody for 1 h. Protein bands were visualized using Enlight Western blotting reagents (Engreen Biosystem, China). The intensities of the bands were quantified using ImageJ. Full blots are included in the supplementary information files (Figs S3–S7).

### Northern blot

Total RNA from HepG2 and HepG2-4D14 cells was extracted using TRIzol reagent. Northern blot was performed according to the manufacturer’s instructions for the NorthernMax-Gly Kit (Invitrogen, USA) as previously described^20^. The probe for SAMD12-AS1 (nt 200–500) was labeled with digoxigenin using the DIG DNA Labeling Kit (Roche, Switzerland), and chemiluminescent detection was performed using the DIG Chemiluminescent Detection Kit (Roche).

### Rapid amplification of cDNA ends (RACE) assay

RACE was performed as previously described^20^ and carried out with SMARTer^TM^ RACE cDNA Amplification Kit (Clontech, USA) following the manufacturer’s instructions. Briefly, cDNA was reverse transcribed from total RNA from HepG2 cells. PCR was performed with specific primers. Then, the products were cloned into the pRACE vector for sequencing. The primers for SAMD12-AS1 were as follows: 5′ primer, 5′-TGATGCGAAACGATATCCTCTTCGATTTG-3′ and 3′ primer, 5′-ACCATAGTTGA TATTCTGAATGGCTTCTGTTCC-3′.

### S1-pulldown and mass spectrometry analysis

HepG2 cells were infected with lentivirus carrying control-S1 or SAMD12-AS1-S1, and then GFP-positive cells were sorted and expanded. The S1 aptamer can bind to streptavidin and be applied to pulldown assays as previously described^33^. Briefly, cells were UV cross-linked and lysed with lysis buffer containing RNase inhibitor for 20 min. The cell lysates were incubated with prewashed Streptavidin T1 magnetic beads (Invitrogen, USA) at 4 °C for 3 h. Then, the magnetic T1 beads were washed 5 times. After washing, the beads were boiled for 15 min in 0.1% SDS and subjected to SDS-PAGE. The proteins in the gel were subjected to mass spectrometry. Data were analyzed using the Mascot search engine and the SwissProt human database.

### Immunoprecipitation

For endogenous NPM1 immunoprecipitation experiments, anti-NPM1 antibody or rabbit IgG (as a control) and protein A beads were used. As previously described^20^, HepG2 cells were UV cross-linked and were lysed with lysis buffer (25 mM Tris-HCl, pH 7.5, 150 mM KCl, 2 mM EDTA, 0.5% NP-40, and 0.5 mM DTT) containing protease inhibitor and RNase inhibitor on ice for 20 min. The lysates were immunoprecipitated with anti-NPM1 antibody or rabbit IgG and protein A beads. The RNA was extracted, and SAMD12-AS1 was quantified by qRT-PCR. For immunoprecipitation of endogenous p53, anti-p53 antibody or mouse IgG (as a control) and protein A beads were used. For endogenous HDM2 immunoprecipitation, anti-HDM2 antibody or rabbit IgG (as a control) and protein A beads were used. For Myc-HDM2 immunoprecipitation, L02 cells were cotransfected with pCMV Myc-HDM2 and control or SAMD12-AS1 plasmids. The cell lysates were harvested for immunoprecipitation with anti-Myc beads. Cell lysates were immunoblotted with the indicated antibodies.

### Dual-luciferase reporter activity assay

HepG2 cells were plated in 24-well plates and cotransfected with the pGL2-SAMD12-AS1-promoter and pRL-TK as an internal control and pCMV FLAG-HBx or pCMV FLAG-HBc. The cell lysates were harvested, and dual-luciferase activity was assessed according to the manufacturer’s instructions (Promega, USA). Relative luciferase activity was calculated (Firefly luciferase/Renilla luciferase).

### CCK8 assay

Cell viability was determined using the CCK8 kit (Dojindo, Japan) according to our previously published method^6^. Briefly, 5000 cells per well were plated into 96-well plates and cultured in DMEM with 1% serum. CCK8 reagent was added at 0, 24, 48 and 72 h and incubated at 37 °C for 1 h. The absorbance was measured at 450 nm using a Microplate Reader (Thermo Fisher, USA).

### Tumor growth assay

A tumor growth assay was performed as described previously^34^. Briefly, 1 × 10^7^ cells were suspended in PBS and injected subcutaneously into 6-week-old BALB/c nude mice. Tumor size was measured and calculated as follows: tumor volume (mm^3^) = (L × W^2^)/2, where L = long axis and W = short axis. Tumor size was measured at each indicated time point. On day 24, the mice were sacrificed, and the tumors were dissected out. All animals received humane care, and the study of mice was permitted by the Research Ethics Committee of the Institute of Microbiology (APZMCAS2016011).

### His-pulldown assay

His pulldown assay was performed according to our previously published method^34^. L02 cells were cotransfected with pCMV His-Ub and with control plasmid or plasmids expressing SAMD12-AS1, SAMD12-AS1(1-350) or SAMD12-AS1(351-701) for 24 h and treated with MG132 (20 μM) for 6 h. The cells were lysed in denaturing lysis buffer at room temperature for 30 min. Then, the cell lysates were mixed with 40 μL of Ni-NTA beads and incubated at room temperature for 4 h. The Ni-NTA beads were washed and eluted with 40 μL of elution buffer (0.15 M Tris-HCl at pH 6.7, 30% glycerol, 5% SDS and 0.5 M imidazole). The solutions were subjected to SDS-PAGE.

### GST pulldown assay

A GST pulldown assay was performed as described previously^30^. Briefly, the GST-HDM2 and His-NPM1 fusion proteins were expressed in *E*. *coli* following induction with IPTG. *In vitro* transcription of RNA for SAMD12-AS1, SAMD12-AS1(1-350) or SAMD12-AS1(351-701) was performed according to the manufacturer’s instructions for the MEGAscript™ T7 Transcription Kit (Invitrogen, USA). Binding was performed in TNE buffer with RNase-free water (140 mM NaCl, 0.5% NP-40, 50 mM Tris-HCl at pH 8.0, 1 mM EDTA, 1 mM PMSF and RNase inhibitor) overnight at 4 °C, and the beads were washed. After washing, the beads were boiled for 15 min in 0.1% SDS and subjected to SDS-PAGE.

### Statistical analysis

The statistical significance between groups was determined using Student’s t-test. P value < 0.05 was considered significant.

## Supplementary information

Supplementary Information
