# Supplementary material for: LncRNA SAMD12-AS1 promotes cell proliferation and inhibits apoptosis by interacting with NPM1
Source: Sci Rep. 2019 Aug 12;9:11593. doi: 10.1038/s41598-019-48116-1 (PMC6691116; doi:10.1038/s41598-019-48116-1)
Supplement: Supplementary file 1 — Supplementary Information [file 41598_2019_48116_MOESM1_ESM.pdf]

# LncRNA SAMD12-AS1 promotes cell proliferation and inhibits apoptosis by interacting with NPM1

Qi Liu[1, 2, 5], Ningning Liu[1], Qilin Shangguan[1, 2], Fang Zhang[1, 2], Wenjia Chai[1, 2], Xiaomei Tong[1], Xin Zhao[3], Zhiwei Li[3], Dandan Qi[1, *], Xin Ye[1, 4, *]

## Supplementary Materials

Supplementary Figures S1-S7

Supplementary Table 1

Supplementary Figure S1: Coding potential prediction of SAMD12-AS1.

a

SAMD12-AS1, long non-coding RNA

| Label | Strand | Frame | Start | Stop | Length (nt \| aa) |
|---|---|---|---|---|---|
| ORF1 | + | 1 | 31 | 141 | 111 \| 36 |
| ORF3 | - | 3 | 528 | 424 | 105 \| 34 |
| ORF2 | - | 1 | 488 | 369 | 93 \| 30 |

b

| Sequence Name | RNA Size | ORF Size | Coding Probability | Coding Label |
|---|---|---|---|---|
| SAMD12-AS1 | 701 | 111 | 0.018391927765621 | no |
| MALAT1 | 8779 | 213 | 0.014395586614209 | no |
| NPM1 | 1449 | 885 | 0.99868791856976 | yes |

a. The ORFs of the SAMD12-AS1 was predicted using ORF Finder (NCBI).

b. Bioinformatic analysis of the coding potential of SAMD12-AS1, MALTA1 (negative control) and NPM1 (positive control) using Coding Potential Assessment Tool (CPAT).

Supplementary Figure S2: The bound proteins identified by mass spectrometry.

| Proteins | Peptide | Score | Expect |
|---|---|---|---|
| NPM1 | GPSSVEDIKAK | 73.58 | 9.10E-07 |
| | GPSSVEDIKAK | 43.24 | 0.00097 |
| | GPSSVEDIKAK | 16.26 | 0.51 |
| | TPKGPSSVEDIK | 13.09 | 0.76 |
| | VDNDENEHQLSLR | 7.72 | 1.4 |
| ANXA2 | QDIAFAYQR | 55.17 | 3.50E-05 |
| | TPAQYDASELK | 34.64 | 0.0049 |
| | TNQELQEINR | 22.73 | 0.095 |
| | AYTNFDAER | 21.19 | 0.035 |
| | VFDRYK | 6.68 | 2.2 |
| HRNR | QGSGSGQSPSR | 50.13 | 6.20E-05 |
| | SSSGSSSSYGQHGSGSR | 46.94 | 3.40E-05 |
| | YGQQGSGSGQSPSR | 31.09 | 0.0044 |
| | SSSRGPYESR | 15.94 | 0.21 |
| ARG1 | GGVEEGPTVLR | 40.9 | 0.00084 |
| | GGVEEGPTVLRK | 4.61 | 4.1 |
| FLG | QSESSHGWTGPSTGVR | 33.61 | 0.0025 |
| | DAVEDLESVGK | 26.64 | 0.027 |
| | GRSAGRSGR | 4.76 | 5.8 |
| | QSGTPHAETSSGGQAASSHEQARS SPGERHGSR | 2.32 | 3.1 |
| RPLP0 | GHLENNPALEK | 32.72 | 0.0087 |
| | LLPHIR | 25.37 | 0.013 |
| | GHLENNPALEK | 22.54 | 0.089 |
| | CFIVGADNVGSK | 15.85 | 0.32 |
| FLNA | GTVEPQLEAR | 26.8 | 0.025 |
| | ANLPQSFQVDTSK | 2.77 | 9.5 |
| DCD | ENAGEDPGLAR | 27 | 0.014 |
| | DAVEDLESVGK | 26.64 | 0.027 |

The cell lysates from HepG2-SAMD12-AS1-S1 and control cells were subjected to S1-pulldown. The bound proteins were resolved in SDS-PAGE and subjected to mass spectrometry. Data were analyzed using the Mascot search engine and the SwissProt human database.

Supplementary Figure S3: Full immunoblotting images for Figure 1f, 1g,1j, 2h and 3h.

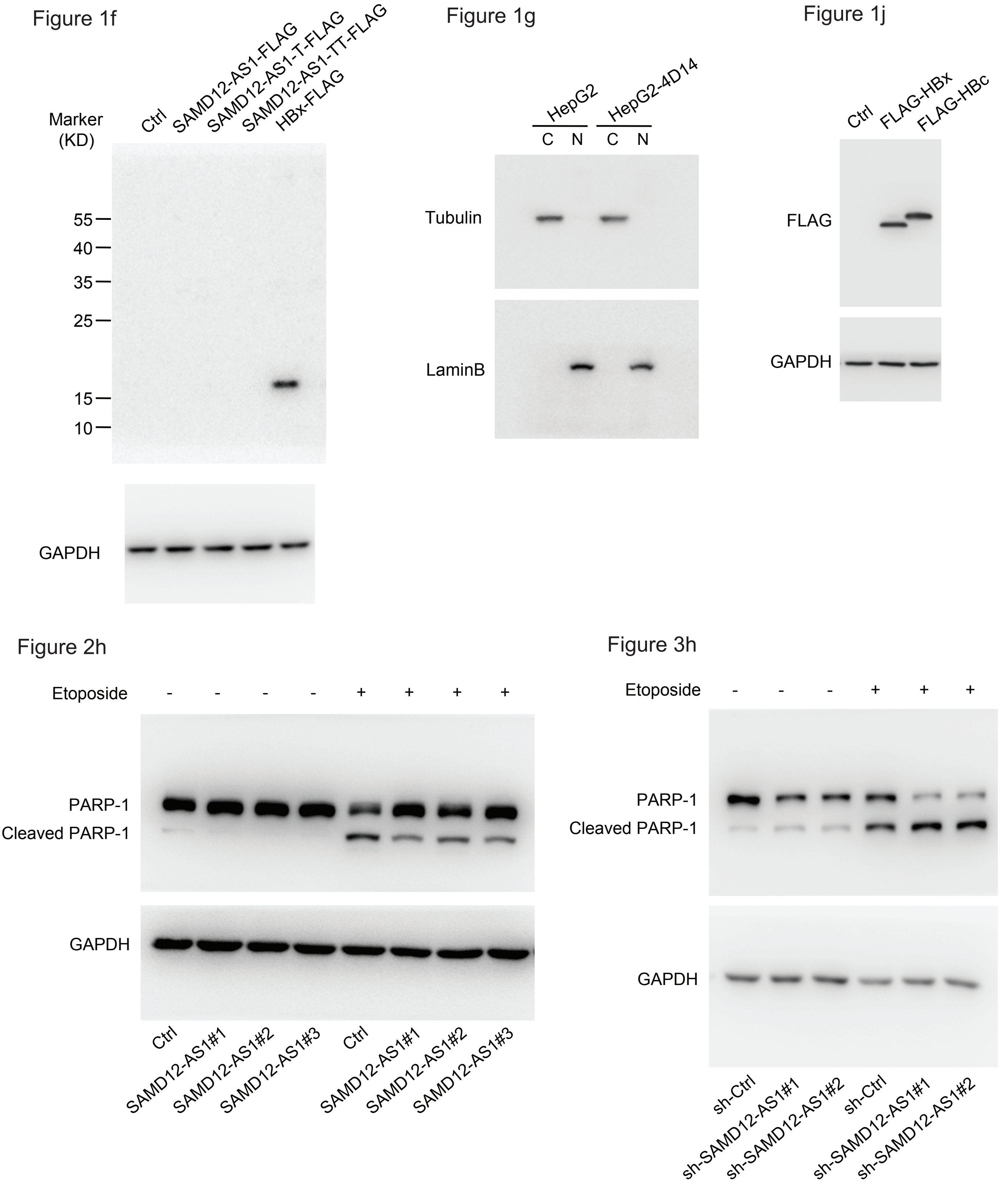

Supplementary Figure S4: Full immunoblotting images for Figure 4.

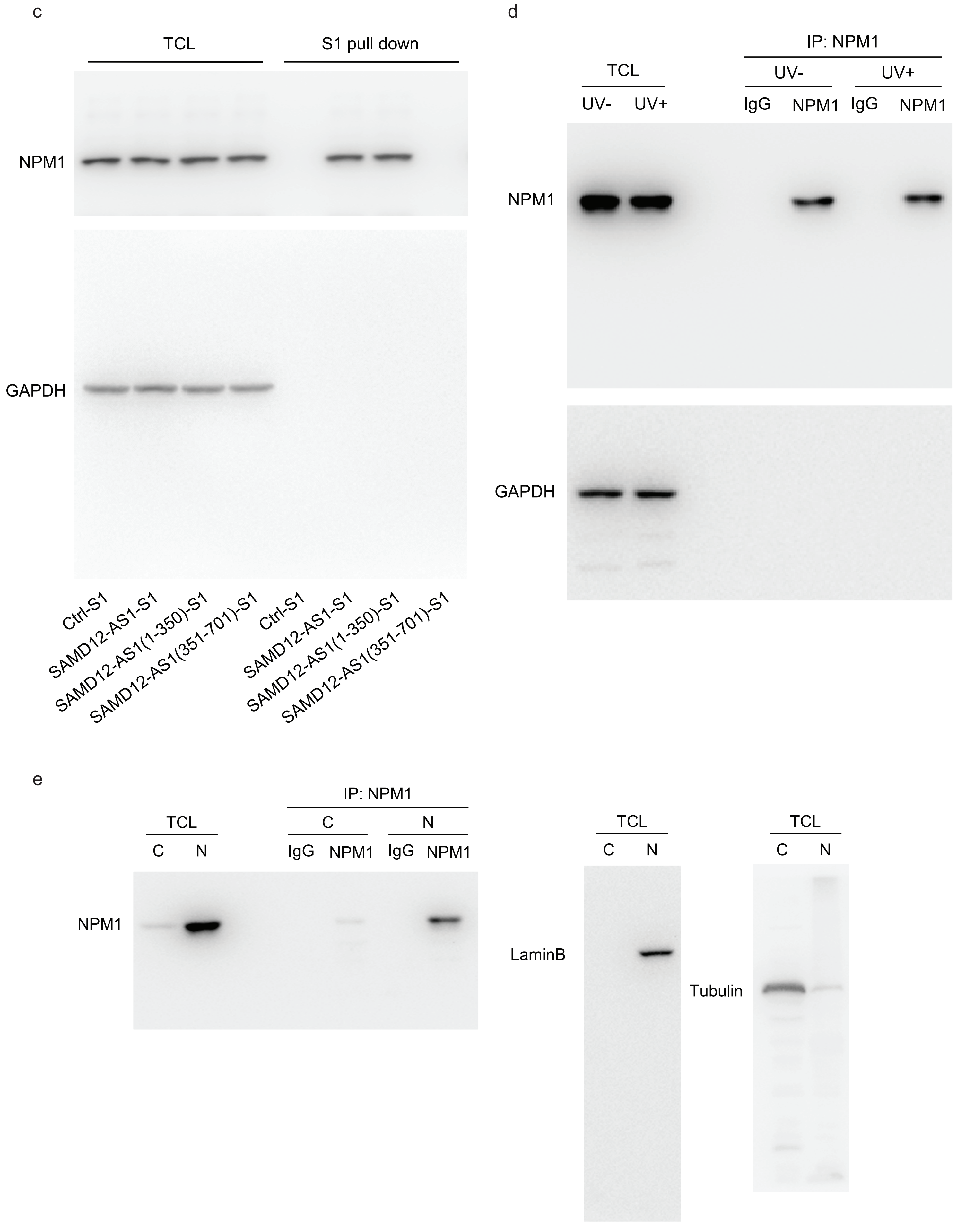

Supplementary Figure S5: Full immunoblotting images for Figure 5.

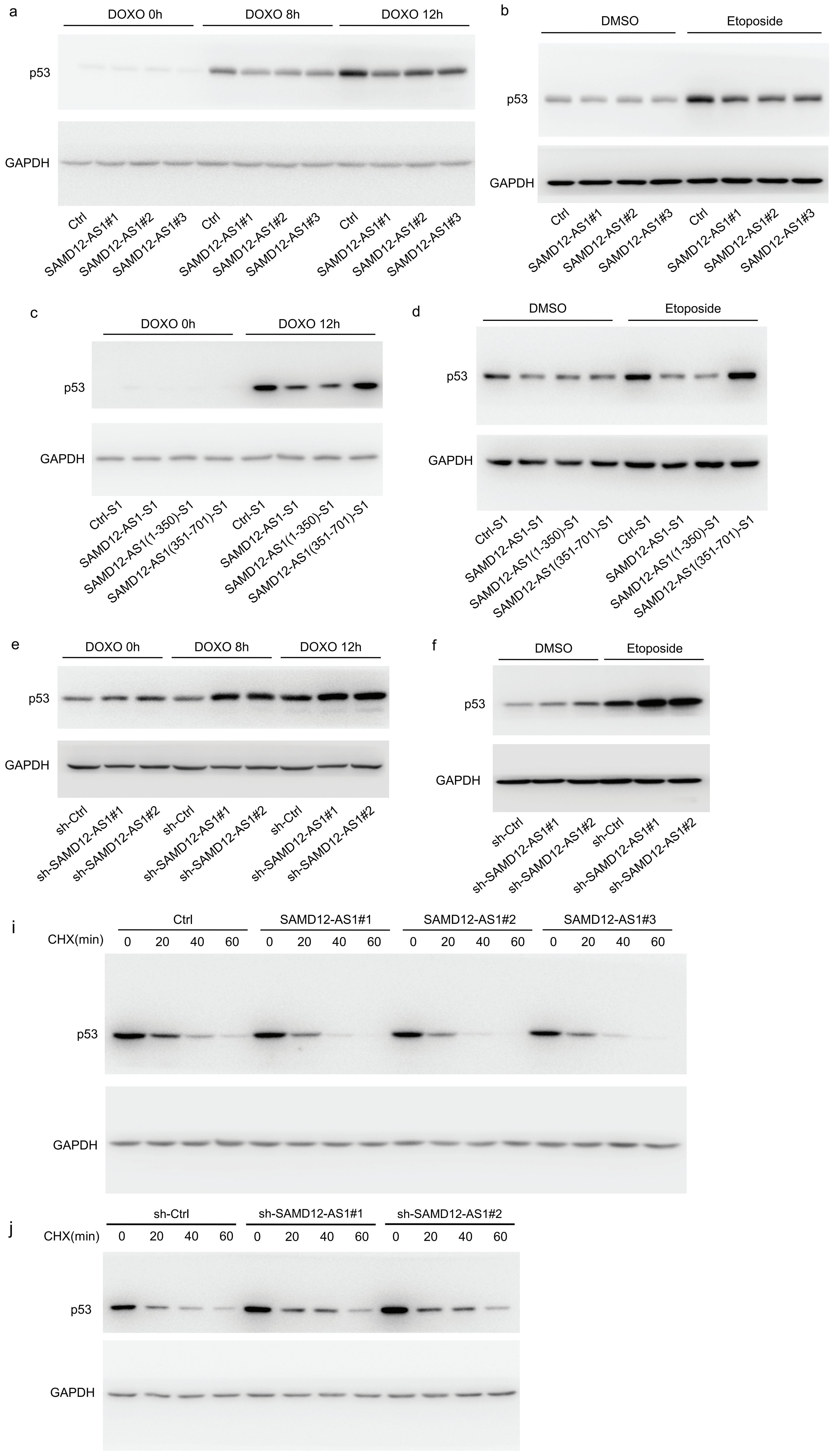

Supplementary Figure S6: Full immunoblotting images for Figure 6.

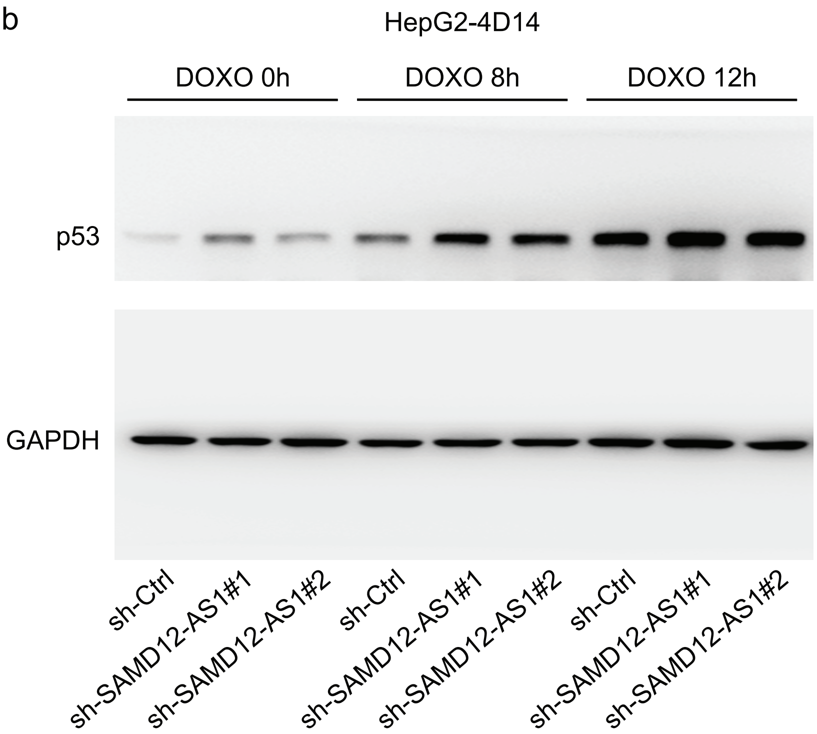


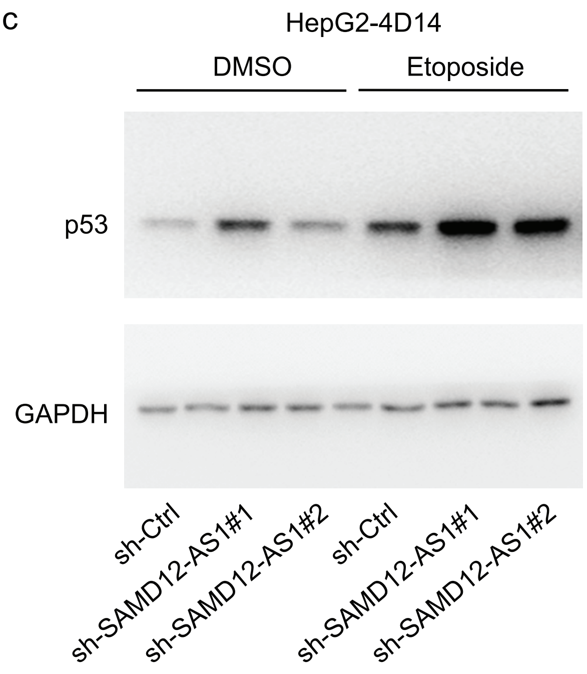


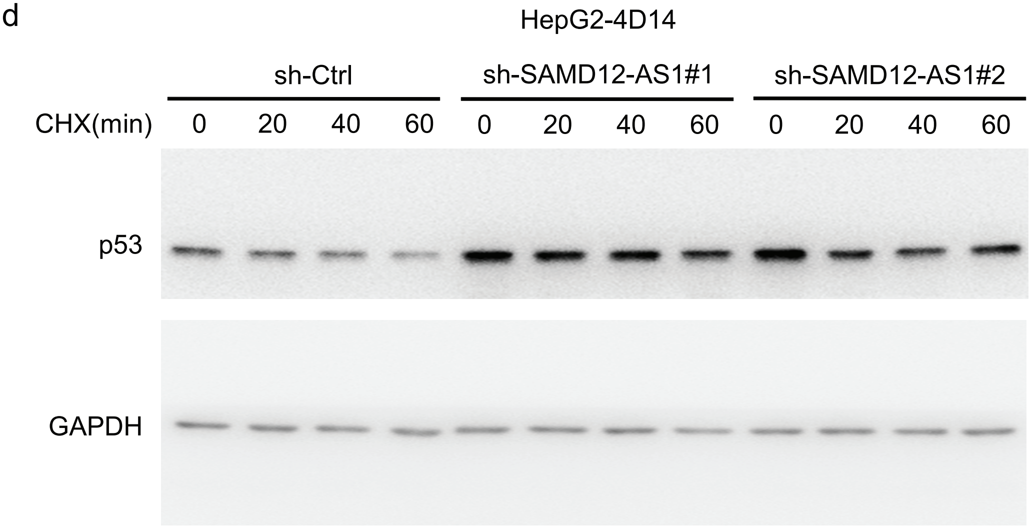

Supplementary Figure S7: Full immunoblotting images for Figure 7.

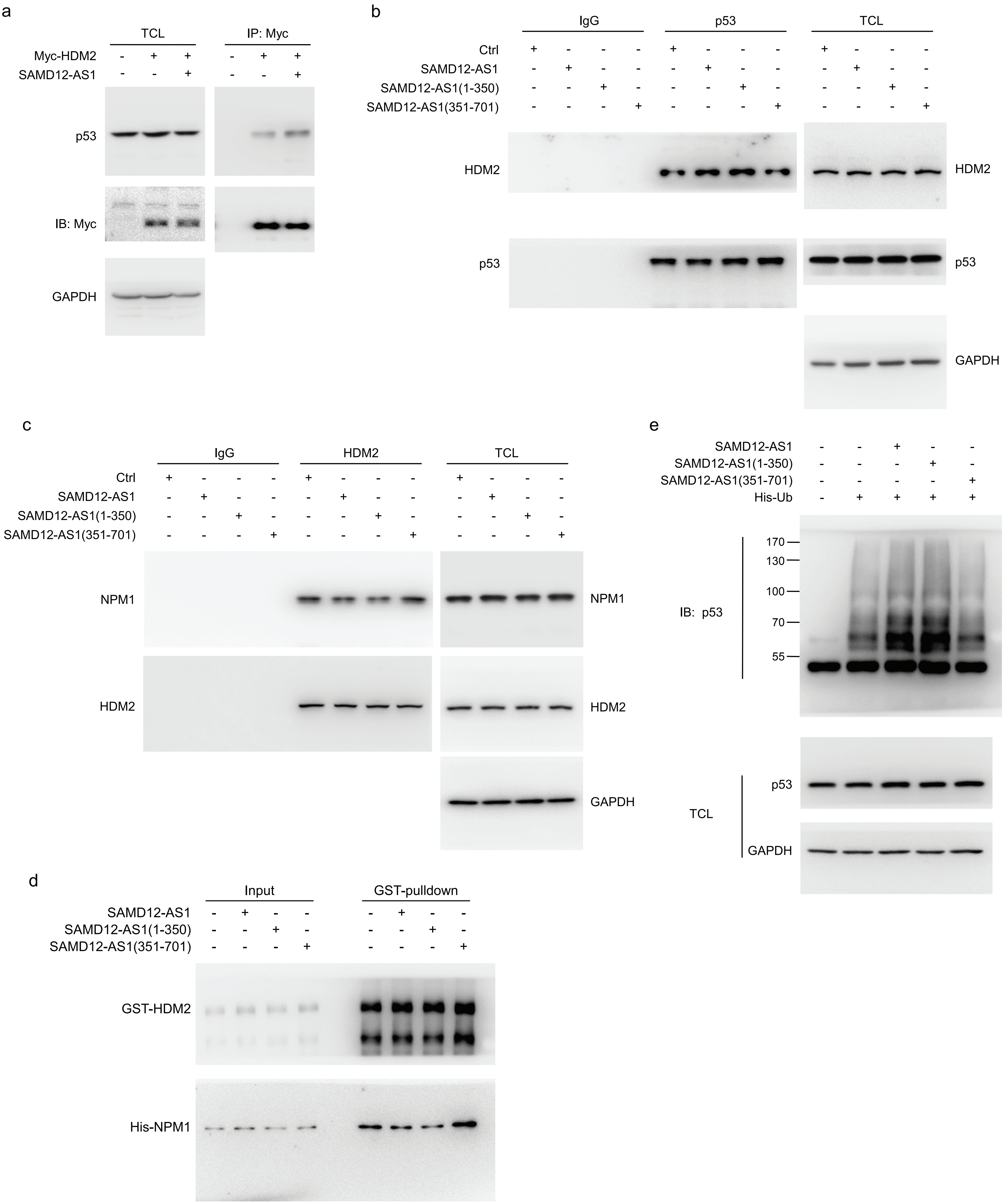

Table 1. Clinical information of 38 paired tissue samples.

| Characteristics | | Number of patients |
|---|---|---|
| Age (year) | ≤50 | 25 |
| | >50 | 13 |
| Gender | Male | 28 |
| | Female | 10 |
| HBsAg | Positive | 19 |
| | Negative | 19 |
| Stage | I | 8 |
| | II | 23 |
| | III | 7 |

HBsAg, Hepatitis B virus surface antigen
